# G-QINDER Tool: Bioinformatically Predicted Formation of Different Four-Stranded DNA Motifs from (GT)_n_ and (GA)_n_ Repeats

**DOI:** 10.3390/ijms24087565

**Published:** 2023-04-20

**Authors:** Lukáš Trizna, Branislav Osif, Viktor Víglaský

**Affiliations:** Department of Biochemistry, Institute of Chemistry, Faculty of Sciences, Pavol Jozef Šafárik University, 04001 Košice, Slovakia; lukas.trizna@yahoo.com (L.T.); branislavsf@me.com (B.O.)

**Keywords:** G-QINDER tool, G-quadruplex, noncanonical DNA structure, tetrahelical motif

## Abstract

The recently introduced semi-orthogonal system of nucleic acid imaging offers a greatly improved method of identifying DNA sequences that are capable of adopting noncanonical structures. This paper uses our newly developed G-QINDER tool to identify specific repeat sequences that adopt unique structural motifs in DNA: TG and AG repeats. The structures were found to adopt a left-handed G-quadruplex form under extreme crowding conditions and a unique tetrahelical motif under certain other conditions. The tetrahelical structure likely consists of stacked AGAG-tetrads but, unlike G-quadruplexes, their stability does not appear to be dependent on the type of monovalent cation present. The occurrence of TG and AG repeats in genomes is not rare, and they are also found frequently in the regulatory regions of nucleic acids, so it is reasonable to assume that putative structural motifs, like other noncanonical forms, could play an important regulatory role in cells. This hypothesis is supported by the structural stability of the AGAG motif; its unfolding can occur even at physiological temperatures since the melting temperature is primarily dependent on the number of AG repeats in the sequence.

## 1. Introduction

Nucleic acids possess the ability to fold into various noncanonical secondary structures that play a fundamental role in the regulation of the physiological processes of cells [1,2,3]. The conformation of a structural motif is generally related to its nucleotide sequence but can also be driven by interactions with molecules in its immediate vicinity. Under certain conditions, specific sequences adopt conformations that are optimal in terms of energy [4]. Some G-rich sequences can fold into highly polymorphic structures known as G-quadruplexes (G4) forming secondary structures consisting of stacked G-tetrad planes connected by a network of hydrogen bonds which are stabilized by monovalent cations such as Na^+^ and K^+^. Changes in the topologies of G4 structures can be induced by, among others, changes in ionic strength, the concentration and composition of salts, temperature, or pH, and also through interactions with small molecules [5]. One of the best examples of a single sequence that can form multiple G4 topologies is human telomeric repeats (HTR). Under experimental conditions, HTR have been found to adopt five well-defined intramolecular G-quadruplexes, four of which consist of three stacked G-tetrads: an antiparallel basket-type in a Na^+^ solution, a parallel crystal propeller form in a K^+^ solution [6,7,8], two different (3 + 1)-hybrid forms in a K^+^ solution, and intramolecular basket-type G4 with only two G-tetrads in a K^+^ solution [9]. On the basis of the identified topological structures and the conditions under which they form, it is clear that the accessibility of water to DNA plays a key role in influencing G4 topology. Dehydrating conditions, also known as crowding conditions, force the telomeric repeats to adopt a parallel G4 structure in a K^+^ solution [10,11,12], and similar conditions can also result in the formation of certain G4-ligand complexes [13]. Interestingly, some putative G4 sequences only form G4 structures when a specific G4 ligand is present in the solution, and the formation of multimeric G4 forms is also dependent on specific conditions. A series of studies examining the same HTR sequence in a K^+^ solution identified the presence of a mixture of several G4 isoforms [14,15,16,17].

**Figure 1:**
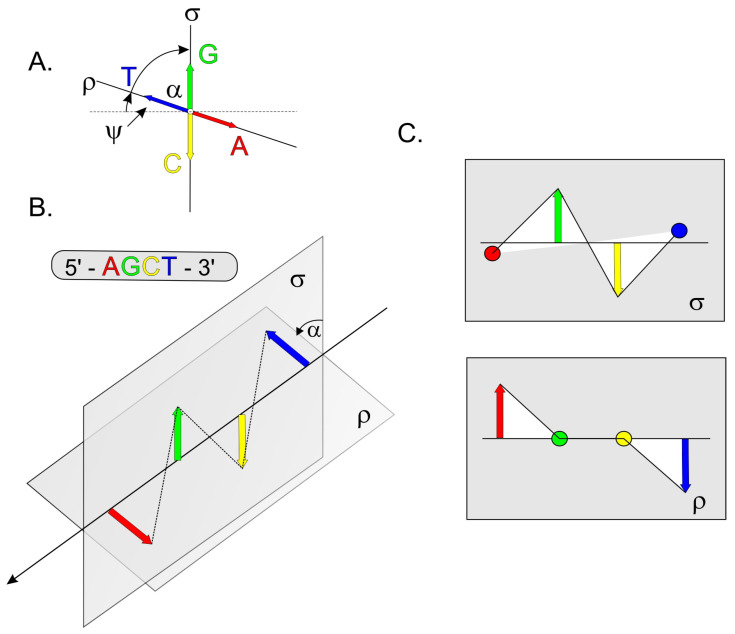
Schematic representation of the semi-orthogonal system (18). (**A**) Orientation of the oligonucleotide in the system; ψ is the angle of inclination from orthogonality, α is the angle of the projection planes of the nucleotides σ and ρ. (**B**) Example of 5′-AGCT-3′ sequence projection in a semi-orthogonal system. (**C**) Projection of this sequence onto the planes σ and r.

A 2022 study by Víglaský described a new method for interpreting nucleic acid sequences called the semi-orthogonal system [18]. Nucleic acid strands consist of a half line on which nucleotides lie at regular distances. The nucleotides are represented by vectors in 3D space perpendicular to a given line, and the orientation of the vector is dependent on the type of nucleotide; the complementary C and G nucleotides lie in one plane, while the A and T nucleotides lie on another perpendicularly opposite plane. However, findings suggest that the optimal angle between the two perpendicular planes for the formation of G4 structures is not 90 degrees but approximately 60 degrees (Figure 1). The projection of the endpoints of the vectors onto each of the planes determines the sequence of nucleic acids, and the line connecting the vertices of the vectors is irregular. The ratio of the area beneath this line to the number of nucleotides offers a useful parameter for quantifying any stretch of nucleic acid sequence and can be used to identify the presence of noncanonical structural motifs. This system, as previously described, allows to predict the adoption of G-quadruplexes and i-motifs in G- and C-rich sequences, respectively, and other secondary motifs, such as the hairpin and cruciform, extruded from perfect and imperfect palindromic repeats.

In this study, this system will be combined with the newly developed G-QINDER tool to identify G-rich sequences not containing two or more Gs adjacent to each other. The freeware version is available at https://biochemistry.science.upjs.sk/g-qinder/index.html (accessed on 12 April 2023). By taking into account the deviations in the perpendicularity of the nucleotide projection planes, different scores were determined for the G3T3, G3T2A, and G3T3 repeats (see Supplementary Table S1). These findings are more accurate than those provided by more commonly used search tools, such as G4Hunter, which offers the same scores for these three sequences [19].

Until recently, it was believed that sequences with fewer than four GG-runs were incapable of forming G4 structures, but the score obtained for d(GT)_n_ predicts the formation of G4 from such repeats at a higher rate than from HTR (Table 1). Once again, other search tools failed to predict the adoption of G4 structures in (GT)_n_ repeats [19,20,21,22,23,24,25,26,27,28,29,30,31].

Longer (GT)_n_ sequences with eight or more repeats occur commonly in various living organisms ranging from bacteria to mammals, and they are also found in some viruses, such as human papillomaviruses (e.g., MT250602.1, KX514421.1, KU298905.1). These types of repeats are found in the human genome, for example, in long intergenic non-protein coding RNA (NR_183720.1), mitochondria (OP682066.1), enhancers (NG_080352.2), and the MHC class II antigen (HLA-DRB1) gene (OP413452.1, OP676256.1). The distribution of longer GT repeats appears to be nonrandom, with these sequences prevalently occurring in noncoding regions, which suggests that these repeats could play important physiological roles [32]. Dinucleotide repeats are ubiquitous features of eukaryotic genomes that are not generally considered to play a functional role in gene expression. However, the high level of variation which they display means that they are likely involved in the modification of RNA splicing in the vicinity of splicing signals. Many studies have demonstrated that UG repeats in RNA are closely associated with different forms of splicing in various gene products, including the human cystic fibrosis transmembrane conductance regulator (CFTR) [33,34,35].

Long sequences of (GA) repeats occur relatively commonly in many organisms, including mammals. Examples of these structures are also found in humans, for example, in lncRNA (NR_183799.1), the MHC class II antigen (DRB3) gene (ON787801.1), and the H3K27ac-H3K4me1 hESC enhancer (NG_143958.1).

Recent studies used NMR spectroscopy to examine an unusual G4 structure that was folded entirely from the r(UG)_12_ sequence (PDB: 7MKT). The authors of [36,37] suggest that similar poly-d(GT) repeats are largely analogous to r(UG)_12_ sequences; therefore, this study aims to verify whether both d(GT)_n_ and d(GA)_n_ sequences are capable of forming G4 structures and noncanonical motifs.

## 2. Results

### 2.1. Analysis of Selected DNA Sequences Using the G-QINDER Tool

To date, the application of the semi-orthogonal system using the G-QINDER tool has identified hundreds of different sequences that are capable of forming G4 structures. The approach assesses the likelihood of the formation of noncanonical motifs by ascribing scores to putative G-rich sequences. Sequences in which the angle α of the projection planes is 60 degrees are ascribed a score greater than 1.2 (Table 1 and Supplementary Table S1).

In this study, however, we focus on sequences without neighboring Gs, which possess either very high G-QINDER scores (Qs) (i.e., Qs > 2) or which lie just below the threshold for G4 motif formation (i.e., 0.7–1.1). As can be seen in Table 1, these types of sequences typically include (GA)_n_ and (GT)_n_ repeats, but it is also interesting to note that sequences adopting Z-G4 structures also show scores higher than 2.

In contrast, several sequences with Qs of less than 1.1 are capable of forming other noncanonical motifs; for example, G3A3, HPV25 [38] adopt the non-G4 tetrahelical VK structure [39,40], as does (GA)_n_. Interestingly, all three of these sequences contain adjacent Gs; studies using NMR assays have not yet confirmed whether G3A3 also forms G4, but preliminary results have suggested that HPV25 does not possess this capacity [38]. In order to clarify the findings of these studies, additional experimental analysis of selected (GA)_n_ and (GT)_n_ repeat sequences has been performed.

### 2.2. CD Analysis of d(GT)_n_ and d(GA)_n_ Sequences

**Figure 2:**
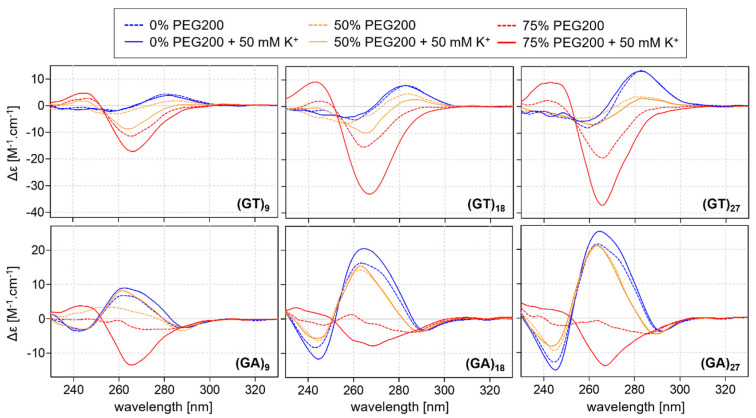
CD spectra of (GT)_n_ and (GA)_n_ in mBR in the presence and absence of 50 mM KCl and pH 7.4 (solid and dashed blue lines, respectively) and with the addition of 50% and 75% PEG200 (red and orange lines, respectively).

The CD spectra results for DNA oligonucleotides with various numbers of GT repeats are shown in Figure 2.

**Figure 3:**
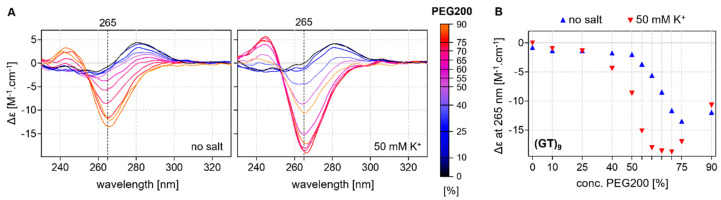
(**A**) CD profiles of (GT)_9_ in the presence and absence of potassium obtained at various concentrations of PEG200; (**B**) Dependence of the negative peak at 265 nm on PEG200 concentration; blue and red triangles represent the values obtained in the absence and presence of 50 mM KCl, respectively.

None of the studied oligonucleotides were found to fold into noncanonical motifs in an aqueous solution, but increased concentrations of PEG200 initiated the formation of a noncanonical motif in sequences with more than eight GT repeats; the clear negative peak in the region of 265 nm in the spectra is similar to that recorded for left-handed Z-G4 structures [41]. While the presence of potassium is known to promote the formation of G4 structures, it is not required to achieve this characteristic profile, and the increase in potassium concentration is not sufficient to produce a G4-like structure without the presence of PEG200. Interestingly, the folding kinetics of the G4 structure were rapid, with the motif forming immediately after the addition of the dehydrating agent PEG, while the formation of Z-G4 structures usually occurs over the course of several days. The effect of PEG200 concentration on the formation of the Z-G4 structure for d(GT)_9_ is shown in Figure 3. The positive influence of potassium on the formation is apparent: G4-like motifs are observed at lower concentrations of PEG200 in the presence of potassium. The dependence of the negative peak at 265 nm on PEG200 suggests that the range of between 60–70% is optimal for Z-G4 formation in a 50 mm KCl solution.

The CD results for (GT)_18_ also indicate the formation of Z-G4 structures (Supporting Figures S1 and S2). A recent study by Das et al. has identified eight GT repeats that are also found in a sequence capable of forming left-handed G4 structures; the formations containing 1–3 bulges consist of two domains, one forming the standard left-handed G4 structure and the other containing bulges (PDB: 7D5D, 7D5E 7D5F) [41]. The left-handed domain is believed to form a skeleton facilitating the formation of a domain with bulges, which also assumes a left-handed orientation. In contrast to these findings, however, our CD observations reveal that sequences in solution with 0–2 bulges initially fold into a right-handed G4 but change their orientation over a longer period of time, although it should be noted that the presence of PEG200 appears to prevent the immediate conversion of these sequences into the left-handed form (results not shown in this study). The CD spectral profile of RNA analog UG repeats is clearly different than those of the d(TG) repeats, but it has been confirmed that this RNA analog adopts a Z-G4 structure [36,37].

The study also analyzed the d(GA)_n_ sequences to investigate the suggestion that the presence of A residues in the loop of G4 structures does not play a significant role in the formation of the motifs. The CD spectra results are shown in Figure 2. Oligonucleotides (GA)_6_ and (GT)_6_, remained in an unfolded state under all of the studied conditions (Supporting Materials, Figure S3). The results also show that (GA)_9_, (GA)_18_, and (GA)_27_ (like (GT)_9_, (GT)_18_, and (GT)_27_) are also capable of adopting Z-G4 structures but only in the presence of potassium and more than 70% PEG200; a negative peak is observed at a wavelength of ~265 nm, although the signal is less intense than those recorded for the (GT)_n_ series. These results suggest that T could be a more suitable base for the formation of Z-G4 motifs than A in these repeat sequences, a hypothesis that was then tested by performing a thermodynamic analysis of the melting curves.

### 2.3. Thermodynamic Stability

**Figure 4:**
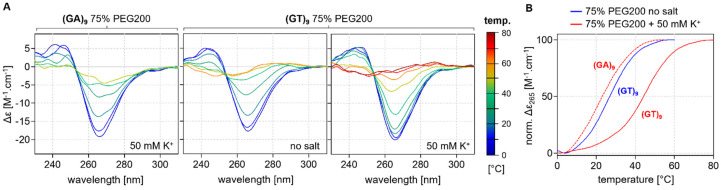
(**A**) CD profiles of (GA)_9_ and (GT)_9_ in the presence and absence of PEG200 obtained at different temperatures with increments of 10 °C; (**B**) Dependence of the negative peak at 265 nm on PEG200 concentration.

CD and UV/Vis melting analyses were performed under the same conditions in order to determine whether the motifs formed from the sequences had a Z-G4 structure; representative results for the (GT)_9_ and (GA)_9_ sequences in the presence of 75% PEG200 are shown in Figure 4. The fading negative CD signal at 265 nm clearly shows that the Z-G4 structure continuously unfolded at increasing temperatures. The stabilizing effect of potassium on the structure formation is also evident. (GA)_9_, (GA)_18_, and (GA)_27_ were not found to form Z-G4 motifs in the absence of potassium, with the melting temperature in the presence of this ion being significantly higher.

The thermodynamic parameters of all of the studied DNA oligonucleotides obtained from the melting curve fitting analysis are summarized in Supplementary Table S2.

**Figure 5:**
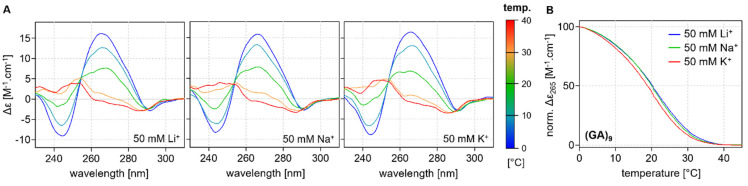
(**A**) CD profiles of (GA)_9_ at various temperatures with increments of 10 °C in the presence of different ions (**B**) Corresponding melting curves obtained at 265 nm: 50 mM LiCl (blue), NaCl (green), and KCl (red).

However, the melting analyses also observed a new phenomenon; the positive CD peaks for (GA)_n_ observed at 265 nm and a slight negative peak at ~290 nm suggest the formation of a new structural motif. At first glance, it might appear that the profile represents a G4 structure, but the formation of the motif is not dependent on the specific type of ion (Figure 5). The stabilization of the G-quartets in all of the G4 structures described to date is dependent on the type of ion, with the highest melting temperatures being recorded in the presence of potassium [10,42]. However, this is not the case with the findings of our analysis because the melting temperatures of the motif were found to be almost identical in the presence of either lithium, sodium, or potassium ions. These spectra suggest that the structure formed from these DNA repeats must be a motif that is distinct from the G4 structure, but it has not been possible to identify its specific form.

The possible existence of a tetrahelical structure formed by the sequence (AG)n was predicted several decades ago before the A-G pairing was confirmed [43,44], and subsequent studies have also proposed the likelihood of an A-G base pairing stabilizing the noncanonical DNA duplex [45]. The CD profile of the (AG)_10_ sequence was analyzed in the past, but the possible formation of a tetrahelical structure was rejected by the authors [46]. Nevertheless, a recent study by Plavec and Kocman using NMR has provided the first direct evidence that AGAG-quartets can contribute significantly to the stabilization of the tetrahelical structure [40].

**Figure 6:**
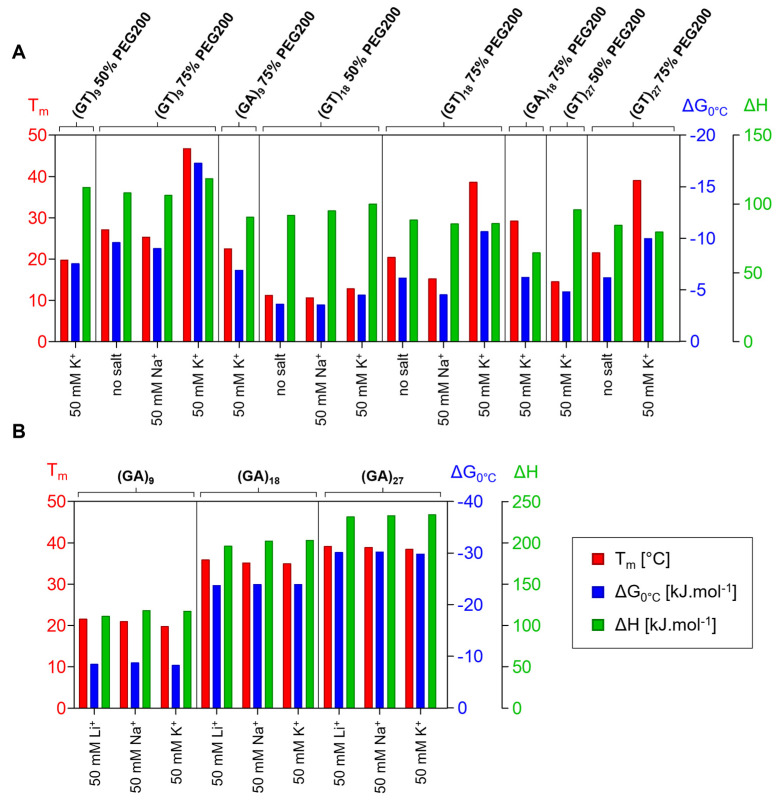
Graphical representation of the data from Supplementary Table S2: melting temperatures (red) and corresponding ΔG calculated at 0 °C (blue) and ΔH (green). The thermodynamic parameters for the Z-G4 structures under molecular crowding conditions are shown in (**A**) and for the AGAG-tetrahelical structures in (**B**).

Under dehydrated conditions, sequences are more likely to fold into G4 motifs than other canonical and noncanonical structures [11,12]. In extreme dehydration conditions where no other motif can form, sequences such as (GA)_n_ and (GT)_n_ can also form less stable G4 structures, but if another noncanonical motif is thermodynamically more stable, then this structure is preferentially formed, as is the case for (GA)_9_, (GA)_18_, and (GA)_27_. In some cases (for example, (GA)_27_), not even the presence of dehydrating agents can force the formation of G4 structures. The thermodynamic parameters clearly show that shorter forms of GT repeats are more stable than longer analogs consisting of more repeats, but the results for the GA repeats suggest that the reverse is the case, with longer (GA)_n_ sequences showing higher stability (Figure 6, and Supporting Table S2 and Figure S5). However, the results also suggest the existence of a critical limit of between six and eight GA and GT repeats for the adoption of Z-G4 and/or tetrahelical motifs.

Interestingly, the dehydrating (crowding) agent PEG200 can be replaced by, for example, PEG400 and 1,2-propanediol; both agents induce a similar effect and can force GA and GT repeats to adopt a Z-G4 structure.

### 2.4. Electrophoretic Analysis

The (GT)_n_ and (GA)_n_ sequences were also analyzed using electrophoresis in order to verify whether the repeats can adopt well-defined structures or a wide range of structural isoforms. One limitation of this assay was the fact that polyacrylamide electrophoretic gels with more than 30% PEG200 could not be produced, and as a result, it was not possible to analyze the Z-G4 motifs under dehydrated conditions. PAGE results in conditions with the presence of different ions are shown in Supplementary Figure S4. As can be seen, no differences in the mobility of the (GT)_n_ sequences were observed under different conditions, and no ionic and temperature dependence was identified. In contrast, the mobility of the (GA)_n_ sequences was found to vary at different temperatures, with the folded and unfolded states also exhibiting different levels of electrophoretic mobility, a property that is crucial for ensuring the continuous monitoring of the temperature dependence of the sample mobility using TGGE [42].

**Figure 7:**
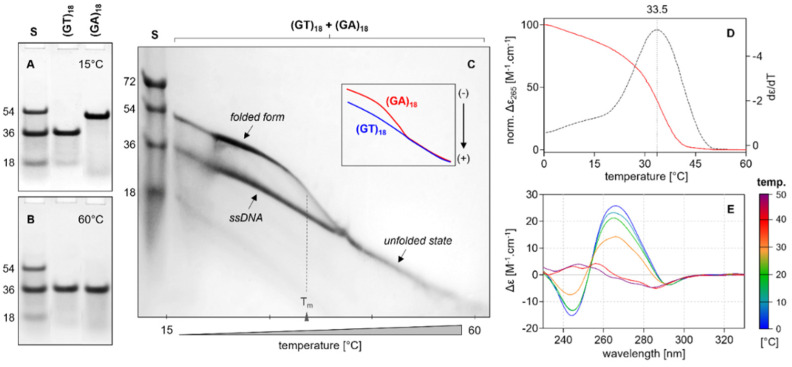
Electrophoretic analysis of (GT)_18_ and (GA)_18_ in mBR in the presence of 50 mM KCl. Electrophoretic records in standard 12% PAGE taken at two different temperatures, (**A**) 15 °C and (**B**) 60 °C, at which DNA occurs in folded and unfolded states, see also Supplementary Figure S4. The temperature of 60 °C is higher than the melting temperature of (GA)_18_. Molecular standard S is a mixture of oligonucleotides d(AC)_9_, d(AC)_18_, and d(AC)_27_. (**C**) Electrophoretic separation of equimolar (GT)_18_ and (GA)_18_ in a temperature gradient perpendicular to electrophoretic motion (TGGE). The results clearly show that the mobility of the dimer (GA)_18_ after melting is equivalent to that of the unstructured (GT)_18_. In order to eliminate unexpected pairing between DNA samples, (GT)_18_ was loaded onto the gel 5 min earlier than (GA)_18_. The inset shows a schematic representation of sample mobilities in the temperature gradient. (**D**) CD melting curve of (GA)_18_ under the same conditions as for PAGE and TGGE (red line) and the first derivative function of the melting curve (dotted line). (**E**) CD spectra collected at different temperatures where the increment is 10 °C.

Figure 7 shows the unfolding of the (GA)_18_ sequence; the melting temperature determined by electrophoresis is in agreement with that obtained through CD spectroscopy, thereby confirming that this oligonucleotide forms a multimeric structure. An increase in electrophoretic mobility was also observed after the unfolding of the sample—an unusual finding given the fact that the mobility of monomers in the unfolded state is usually lower than in the folded state. The electrophoretic mobility of (GA)_27_ also corresponds to that of a multimeric structure (Supporting Figure S4).

### 2.5. Interactions between Noncanonical Motifs and Thiazole Orange

The thiazole orange (TO) ligand is known to interact with nucleic acids and induces a CD signal (ICD) in the visible region. Despite its poor structural selectivity, the ICD of the DNA–TO complex exhibits a specific profile depending on the topology of the DNA structure [13]. Furthermore, if, under certain conditions, the oligonucleotide does not adopt the 3D structure, TO can facilitate or force the oligonucleotide to adopt the formation.

**Figure 8:**
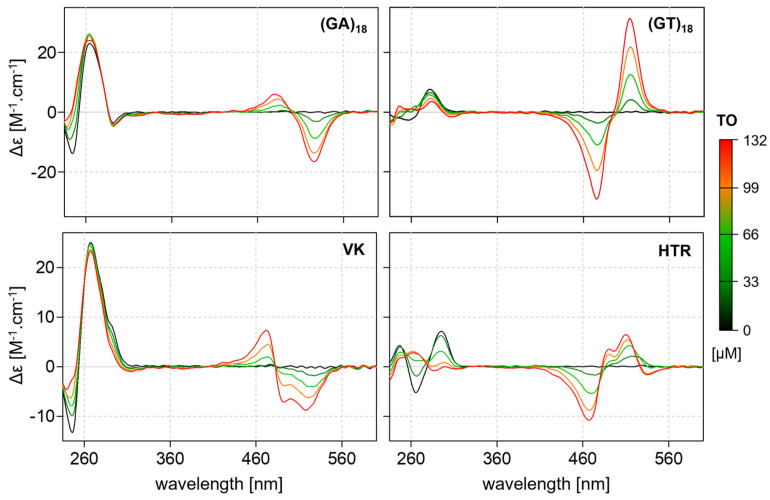
CD spectra of (GA)_18_ and (GT)_18_ in the presence of different concentrations of TO (top) and analogous CD spectra of VK and HTR sequences under the same conditions (bottom). DNA sample concentration is 25 µM and the TO increment is 33 µM.

Figure 8 shows the CD spectra profiles of the complexes of (GA)_18_ and (GT)_18_ with TO in the presence of sodium. The ICD of the (GA)_n_–TO complex is similar to the characteristic profile of tetrahelical VK–TO complexes, while the ICD of the (GT)_n_ complex more closely resembles that identified for G4-TO complexes. Indirect experimental results also indicate that the structure of (GA)_n_ shares some common attributes with the VK motif. The CD spectra of (GT)_n_ are in agreement with the recently proposed Z-G4 structure [39,40].

### 2.6. Structural Model of (AG)_n_

**Figure 9:**
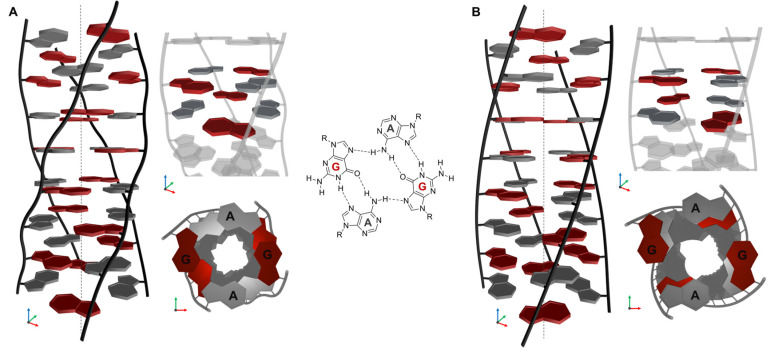
(**A**) Proposed noncanonical motif based on analogies with the noncanonical tetrahedral VK structure, in which the guanines in one AGAG-quartet are oriented in syn- and in anti-conformation in the adjacent quartet. The core of the structure is formed by unusual AGAG-quartets stacked on top of each other. A schematic representation of the AGAG-quartet is shown in the middle. (**B**) Structural model in which all guanines are oriented in the syn-conformation. The schematic representation of the structures was created in the Blender 3.5 freeware modeling tool.

AGAG-quartets are known to feature in the tetrahelical VK motif [39,40], and we, therefore, assume that these structures may also contribute to the stability of the motifs observed in our studies given their similarity. The structural motif is not dependent on the type of monovalent cations [40], but G-C pairing has not been observed in the case of (GA)_n_ sequences, which suggests that the observed structure consists only of AGAG-quartets. The hypothetical proposed structures are shown in Figure 9. In these models, the guanines in the AGAG-quartets are assumed to be either in the anti-conformation or in both the anti- and syn-conformations. The stacking of these quartets seems to be more effective in the case of alternating conformations of the guanines. Our results confirm that six repetitions of GA are still insufficient to form such a motif, but that formation can occur in the case of nine or more repeats. Based on the results of the CD spectroscopy, electrophoresis, and titration experiments with TO, and also on the basis of observations published by other authors, we hypothesize that the core of the noncanonical (AG)_n_ structure consists of AGAG-quartets. We also assume that more than 12 quartets are present in the case of the tetrameric structure since the CD spectrum of (GA)_6_ did not indicate the formation of this type of structure, but it should be noted that (GA)_9_ can adopt this structural motif even without the presence of a monovalent cation. Although the lower mobility revealed in the electrophoretic analysis suggests that the structure could be a multimeric form in the folded state, this hypothesis cannot be confirmed on the basis of electrophoresis alone. The mobility of the folded structure in the case of the (GA)_9_, (GA)_18_, and (GA)_27_ sequences is lower than that of the unfolded structure, suggesting that the folded state could be a multimer (Figure 6 and Supplementary Figure S4). We also suggest that the band migrating more slowly at 15 °C is representative of a tetrameric rather than a dimeric structure, and although the possibility of a dimeric form cannot be completely ruled out, at least in the case of (GA)_27_ (Supplementary Figure S4), this is one of the simplest explanations for our hypothesis of a tetrahelical structure. This claim is supported by the results obtained using Mung Bean nuclease; this enzyme preferentially digests (GT)_18_ and VK but does not change (GA)_18_ (Supplementary Figure S6). (GT)_18_ does not form a secondary structure, presumably occurring in the unfolded single-stranded form at a given condition, and the VK structure contains loops recognized by this enzyme. If (GA)_n_ sequences adopted a dimeric conformation, then loops would be present and we would expect to observe their cleavage, but no shorter fragments were detected in the electrophoretic analyses performed at temperatures above the melting temperatures.

The orientation of the DNA strands in the proposed structure as well as the overall structure require verification by other means, including NMR, but it is clear from our results that the (AG)_n_ sequences with eight or more repeats form a noncanonical structural motif. This motif cannot be a G-quadruplex because it is not stabilized by potassium cations; it also shares some common features with the VK motif.

## 3. Materials and Methods

All experiments were carried out in a modified Britton–Robinson buffer (mBR) using 25 mM phosphoric acid, 25 mM boric acid, and 25 mM acetic acid. KCl, NaCl, or LiCl was added to the solutions; the final concentration was 50 mM, and the pH was adjusted to a final value of 7.0 using Tris. A dehydrating condition was adjusted with PEG200 (polyethylene glycol with an average molecular weight of 200) (Fisher Slovakia, Bratislava, Slovakia). Oligonucleotides with the sequences shown in Table 1 were purchased from Metabion International AG. The lyophilized DNA samples were dissolved in double-distilled water prior to use to yield 1 mM stock solutions. Single-strand DNA concentrations were determined by measuring the absorbance at 260 nm at a high temperature (95 °C).

This study was the first to use the G-QINDER tool developed on the basis of the recently described semi-orthogonal system. Freeware versions for Windows and MacOS are available at https://biochemistry.science.upjs.sk/g-qinder/index.html (accessed on 12 April 2023).

### 3.1. Circular Dichroism Spectroscopy

CD spectra were measured using a Jasco J-810 spectropolarimeter equipped with a PTC-423L temperature controller. The DNA sample was analyzed in a 1 mm quartz cell, and the reaction volume was ~150 µL; the scanning speed of the instrument was set at 100 nm/min, 1 nm pitch, and 1 nm bandwidth, with a response time of 2 s. CD spectra represent an average of three scans taken at a temperature range of 0–100 °C. Scans were performed over a range of 220–350 nm and 220–700 nm in the presence of thiazole orange (TO). All other parameters and conditions were identical to those in the method described previously [13].

#### CD Melting Curves

CD melting profiles were collected at ~265 as a function of temperature using the previously published procedure [13,47]. The temperatures ranged from 0 to 100 °C, and the heating rate was 0.5 °C per minute. The melting temperature (T_m_) was estimated as the temperature of the mid-transition point and was determined using a fitting analysis of the two-state system. DNA titration was performed with increasing concentrations of TO. TO was solubilized in DMSO to reach a final concentration of 10 mM. The concentration of DNA and TO in the 1 mm quartz cells was 25 μM and 0–200 μM, respectively, and the increment of TO was ~33 μM. Each sample was mixed vigorously for 3 min following the addition of TO; CD/UV spectra were then measured immediately [13].

### 3.2. Electrophoresis

Samples consisting of ~0.3 µL of 1 mM stock solutions were separated using nondenaturing PAGE in a temperature-controlled electrophoretic apparatus (Z375039-1EA; Sigma-Aldrich, San Francisco, CA, USA) on 12% acrylamide (19: 1 acrylamide/bisacrylamide) gels. Electrophoresis was run at 15° and 60°C for 2 h at 125 V (~8 V·cm^−1^). All electrophoretic measurements were performed in the mBR buffer at pH 7.0. Temperature gradient gel electrophoresis (TGGE) equipment was used according to the previously described method [42,47]. As in the previous study, the gel concentration was 12%. Electrophoreses were run perpendicularly to the temperature gradient (15–80 °C) for 3 h at 160 V (~8 V·cm^−1^). Approximately 12 μg of DNA was loaded into the electrophoretic well. Each gel was stained with StainsAll (Sigma-Aldrich).

## 4. Conclusions

As was mentioned in the introduction, the repeat sequences examined in this study occur quite frequently in the genomes of different organisms; therefore, a greater understanding of the formation of noncanonical motifs could play an important role in gene expression, RNA editing, or DNA recombination. In this study, we analyzed a series of (GA)_n_ and (GT)_n_ repeat sequences and applied the new G-QINDER tool that predicts the formation of noncanonical structural motifs, including G-quadruplexes and i-motifs. Other approaches using a different strategy to identify such motifs have distinct disadvantages that render them less suitable for the analysis of the sequences investigated in this study. The semi-orthogonal presentation of nucleic acid sequences offers a rational explanation for why, for example, the G4Hunter algorithm fails to predict noncanonical motifs formed from sequences that, although rich in Gs, are interrupted by other nucleotides; the G4Hunter algorithm’s score is below the threshold for predicting G4 structure. Moreover, as we have shown in this study, such a noncanonical structure is not necessarily a G4 motif; thus, false positives are greatly reduced. Therefore, we believe that the G-QUINDER tool will be valuable for researchers focusing on the study of noncanonical nucleic acid structures.

The results offer the surprising finding that d(GT)_n_ in dehydrated conditions adopts not a conventional G4 structure but a Z-G4 form. At present, it is not clear whether such extreme conditions can occur in living cells, but it is possible that this could arise under specific stress conditions or in extremophile organisms. If this were the case, we would expect that Z-G4 DNA could also play an important biological role in addition to that of the RNA structures. It should be noted that the Z-G4 motif is typically formed in a crystal form under conditions in which there is limited access to free water from the solvent to the DNA [41].

In contrast to the (GT)_n_ sequence, the (GA)_n_ sequence with alternating purines has been the subject of several previous scientific studies [43,44,45,46], but the structure of the motif still remains unclear. Some studies assume that the form adopts a parallel-oriented dsDNA, while others suggest that it forms an antiparallel structure, and the possibility of a tetraplex formation has also been proposed. Nonetheless, all the existing studies are in agreement that an alternative pairing between adenines and guanosines must be involved in the proposed noncanonical motif.

Another unanswered question relates to the issue of whether the RNA analog r(GA)_n_ also forms similar noncanonical structures. It is known that RNA tends to orient guanosines into a syn-conformation in contrast to the anti-conformation, which is preferred by DNA. In RNA, most syn nucleobases participate in tertiary stacking and base-pairing interactions [48]. Nevertheless, we believe that one of the models proposed in this study would also be applicable to the structure of the RNA analog, but the full structure of this interesting structure will need to be determined before this issue can be addressed.

## Figures and Tables

**Table 1:** DNA sequences analyzed in the study. Sequences highlighted in pale orange and green possess Qs greater than 2 or lie at the interval (0.7–1.1), respectively.

Name	DNA Sequence	nts	Qs	
			Ψ = 15°	30°
(TG)_6_	GTGTGTGTGTGT	12	1.73	2.06
(TG)_9_	GTGTGTGTGTGTGTGTGT	18	1.78	2.12
(TG)_18_	GTGTGTGTGTGTGTGTGTGTGTGTGTGTGTGTGTGT	36	1.84	2.18
(TG)_27_	GTGTGTGTGTGTGTGTGTGTGTGTGTGTGTGTGTGTGTGTGTGTGTGTGTGTGT	54	1.85	2.20
(GA)_6_	GAGAGAGAGAGA	12	1.02	0.69
(GA)_9_	GAGAGAGAGAGAGAGAGA	18	1.05	0.71
(GA)_18_	GAGAGAGAGAGAGAGAGAGAGAGAGAGAGAGAGAGA	36	1.08	0.73
(GA)_27_	GAGAGAGAGAGAGAGAGAGAGAGAGAGAGAGAGAGAGAGAGAGAGAGAGAGAGA	54	1.09	0.74
VK	GGGAGCGAGGGAGCGAGGGAGCGAGGGAGCG	31	1.28	1.11

## Data Availability

The data presented in this study are available in the article and Supplementary Materials.

## Supplementary Materials

The supporting information can be downloaded at: https://www.mdpi.com/article/10.3390/ijms24087565/s1.
