# Supplementary material for: G-QINDER Tool: Bioinformatically Predicted Formation of Different Four-Stranded DNA Motifs from (GT)n and (GA)n Repeats
Source: Int J Mol Sci. 2023 Apr 20;24(8):7565. doi: 10.3390/ijms24087565 (PMC10198322; doi:10.3390/ijms24087565)
Supplement: Supplementary file 1 [file ijms-24-07565-s001.zip › ijms-2349364-supplementary.pdf]

International Journal of *Molecular Sciences*

MDPI

Article

G-QINDER Tool: Bioinformatically Predicted Formation of Different Four-Stranded DNA Motifs from $(GT)_n$ and $(GA)_n$ Repeats

Lukáš Trizna, Branislav Osif and Viktor Víglaský *

Department of Biochemistry, Institute of Chemistry, Faculty of Sciences, Pavol Jozef Šafárik University, 04001 Košice, Slovakia; lukas.trizna@yahoo.com (L.T.); branislavsf@me.com (B.O.)
* Correspondence: viktor.viglasky@upjs.sk; Tel.: +421-55-2341262

G-QINDER freeware versions for Windows and MacOS are available at https://biochemistry.science.upjs.sk/g-qinder/index.html. Details regarding the source code are also available at https://github.com/branis97/G-QINDER.

Table S1. G-QINDER results obtained for different G-rich sequences. Sequences highlighted in light blue, and orange represent sequences which adopt G4 and Z-G4 motifs, respectively. The sequence highlighted in pale green with Qs ranging from 0.6-1.1 may adopt a non-canonical motif other than G4. Sequences $d(GT)_6$ and $d(GA)_6$ are too short to form any secondary structure; both occur in an unfolded state, but their longer derivatives can adopt non-canonical motifs.

Abbreviation	Sequence	nts	Q-Score 30°
**TBA**	GGTTGGTGTGGTTGG	15	**2.20**
**c-MYC**	TGGGGAGGGTGGGGAGGGTGGGGAAGG	27	**2.08**
**HTR**	GGGTTAGGGTTAGGGTTAGGG	21	**1.79**
**A-HTR-T**	AGGGTTAGGGTTAGGGTTAGGGT	23	**1.76**
**G3T**	GGGTGGGTGGGTGGG	15	**2.50**
**G3A**	GGGAGGGAGGGAGGG	15	**1.90**
**G3T2**	GGGTTGGGTTGGGTTGGG	18	**2.33**
**G3A2**	GGGAAGGGAAGGGAAGGG	18	**1.33**
**G3T3**	GGGTTTGGGTTTGGGTTTGGG	21	**2.21**
**G3A3**	GGGAAAGGGAAAGGGAAAGGG	21	**0.93**
**G3T4**	GGGTTTTGGGTTTTGGGTTTTGGG	24	**2.12**
**G3A4**	GGGAAAAGGGAAAAGGGAAAAGGG	24	**0.63**
**G4T**	GGGGTGGGGTGGGGTGGGG	19	**2.61**
**G4A**	GGGGAGGGGAGGGGAGGGG	19	**2.13**
**G4T2**	GGGGTTGGGGTTGGGGTTGGGG	22	**2.45**
**G4A2**	GGGGAAGGGGAAGGGGAAGGGG	22	**1.64**
**G4T3**	GGGGTTTGGGGTTTGGGGTTTGGGG	25	**2.34**

**G4A3**	GGGGAAAGGGGAAAGGGGAAAGGGG	25	**1.26**
**G4T4**	GGGGTTTTGGGGTTTTGGGGTTTTGGGG	28	**2.25**
**G4A4**	GGGGAAAAGGGGAAAAGGGGAAAAGGGG	28	**0.96**
**Z-G4**	TGGTGGTGGTGGTTGTGGTGGTGGTGT	27	**2.33**
**Z-G4m**	TGGTGGTGGTGGTGTGGTGGTGGTGT	26	**2.37**
**Z-G4-A**	TGGAGGAGGAGGAGAGGAGGAGGAGT	26	**1.44**
**GAG9**	AGAGGAGGAGGAGGAGGAGGAGGAGGAGA	29	**1.34**
**Z-G4-1xbulge**	GGTGTGTGGTGGTGTGGTGGTGGTG	25	**2.34**
**-2xbulge**	GGTGTGTGTGTGGTGTGGTGGTGGTG	26	**2.31**
**-3xbulge**	GGTGTGTGTGTGTGTGTGGTGGTGGTG	27	**2.28**
**-4xbulge**	GTGTGTGTGTGTGTGTGTGGTGGTGGTG	28	**2.25**
**(GGTT)$_8$**	GGTTGGTTGGTTGGTTGGTTGGTTGGTTGGTT	32	**2.18**
**(GGAA)$_8$**	GGAAGGAAGGAAGGAAGGAAGGAAGGAAGGAA	32	**0.73**
**(GT)$_6$**	GTGTGTGTGTGT	12	**2.06**
**(GT)$_9$**	GTGTGTGTGTGTGTGTGT	18	**2.12**
**(GT)$_{18}$**	GTGTGTGTGTGTGTGTGTGTGTGTGTGTGTGTGTGT	36	**2.18**
**(GT)$_{27}$**	GTGTGTGTGTGTGTGTGTGTGTGTGTGTGTGTGTGTGTGTGTGTGTGTGTGTGT	54	**2.20**
**(GA)$_6$**	GAGAGAGAGAGA	12	**0.69**
**(GA)$_9$**	GAGAGAGAGAGAGAGAGA	18	**0.71**
**(GA)$_{18}$**	GAGAGAGAGAGAGAGAGAGAGAGAGAGAGAGAGAGA	36	**0.73**
**(GA)$_{27}$**	GAGAGAGAGAGAGAGAGAGAGAGAGAGAGAGAGAGAGAGAGAGAGAGAGAGAG A	54	**0.74**
**VK**	GGGAGCGAGGGAGCGAGGGAGCGAGGGAGCG	31	**1.11**
**VK-1/2**	GGGAGCGAGGGAGCG	15	**1.10**
**HPV-25-1**	GGGAGCGGGACTGGGACCGGGACCGGGA	28	**0.83**
**HPV-25-2**	GGGAGCGGGACTGGGACCGGGA	22	**0.99**
**HPV-25-3**	GGGACTGGGACCGGGACCGGGA	22	**0.72**

Table S2. Thermodynamic parameters of GA- and GT-repeats. The accuracy of the Tm values is ± 0.6 °C, the accuracy of the parameters ΔH and ΔG (at 0 and 20 °C) was determined with a deviation of 5 %. Parameters corresponding to the tetrahelical motif are highlighted in light blue, other values correspond to the Z-G4 motif.

oligo	conc. PEG200	salt [mM]	T_m [°C]	ΔH [kJ.mol^{-1}]	ΔG (0°C) [kJ.mol^{-1}]	ΔG (20°C) [kJ.mol^{-1}]
$(GT)_9$	50%	-	ND	ND	ND	ND
		50 K^+	19.7	112.0	-7.5	0.1
	75%	-	27.1	108.0	-9.6	-2.6
			25.3	106.2	-9.0	-1.9
		50 K^+	46.7	118.3	-17.3	-9.9
$(GA)_9$	-	50 Li^+	21.5	115.6	-8.4	-0.6
		50 Na^+	20.9	122.8	-8.7	-0.4
		50 K^+	19.7	122.1	-8.2	0.1
	75%	-	ND	ND	ND	ND
		50 K^+	22.5	90.4	-6.9	-0.8
$(GT)_{18}$	50%	-	11.2	91.7	-3.6	2.8
		50 Na^+	10.6	95.0	-3.5	3.2
		50 K^+	12.8	100.0	-4.5	2.5
	75%	-	20.4	88.3	-6.1	-0.1
		50 Na^+	15.2	85.5	-4.5	1.4
		50 K^+	38.6	85.8	-10.6	-5.1
$(GA)_{18}$	-	50 Li^+	35.9	203.8	-23.7	-10.5
		50 Na^+	35.1	210.3	-23.9	-10.3
		50 K^+	34.9	211.3	-23.9	-10.2
	75%	-	ND	ND	ND	ND
		50 K^+	29.2	64.6	-6.2	-2.0
$(GT)_{27}$	50%	-	ND	ND	ND	ND
		50 K^+	14.5	95.7	-4.8	1.8
	75%	-	21.5	84.5	-6.2	-0.4
		50 K^+	39.0	79.6	-9.9	-4.8
$(GA)_{27}$	-	50 Li^+	39.1	240.7	-30.1	-14.7
		50 Na^+	38.9	242.4	-30.2	-14.7
		50 K^+	38.4	243.3	-29.8	-14.2

ND - not determined; secondary structure is not formed

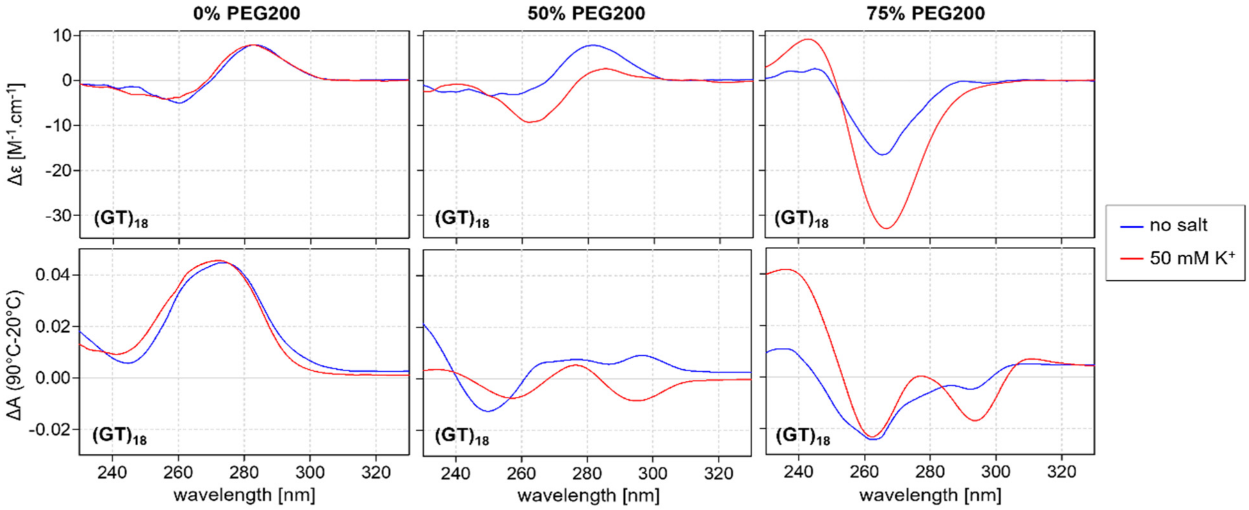

Figure S1. CD spectra of (GT)18 (top) and corresponding UV/Vis thermal difference spectra (TDS -below) at different concentrations of PEG200 in the absence of potassium (blue lines) and 50 mM KCl (red lines)

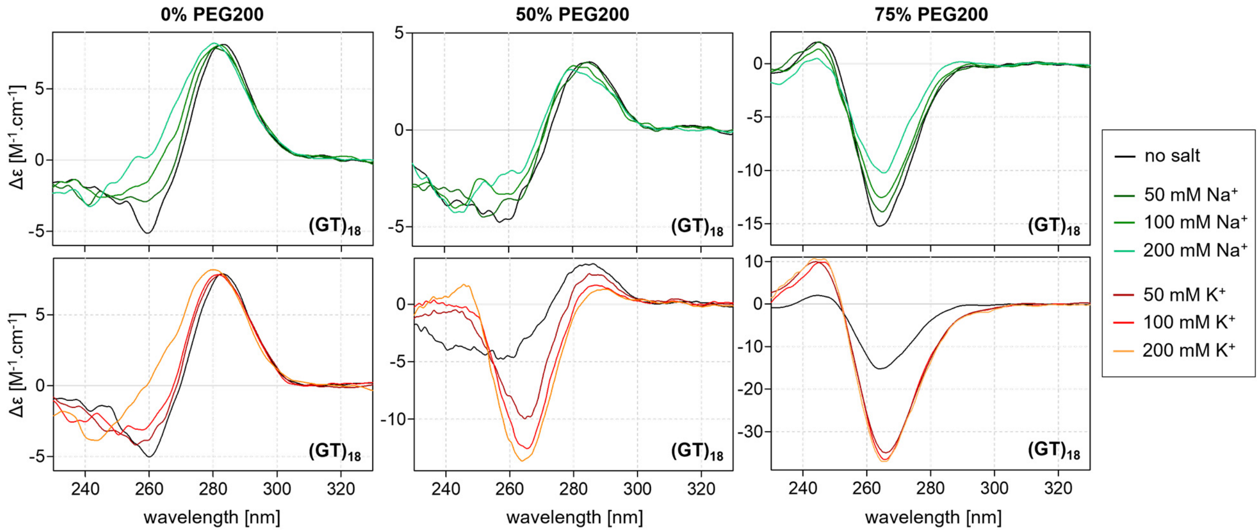

Figure S2. CD spectra of (GT)18 at various concentrations of PEG200 and different salts.

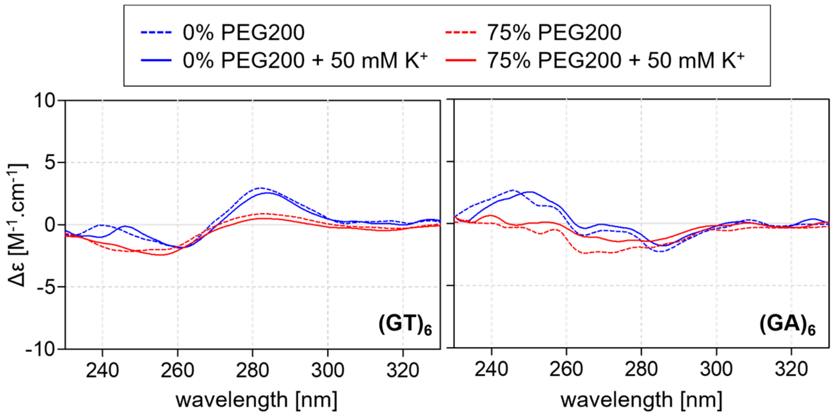

Figure S3. CD spectra of (GT)₆ and (GA)₆ in mBR in the presence and absence of 50 mM KCl, pH 7.4 (solid and dashed blue lines, respectively) and in the presence of 50% and 75% PEG200 (red and orange lines).

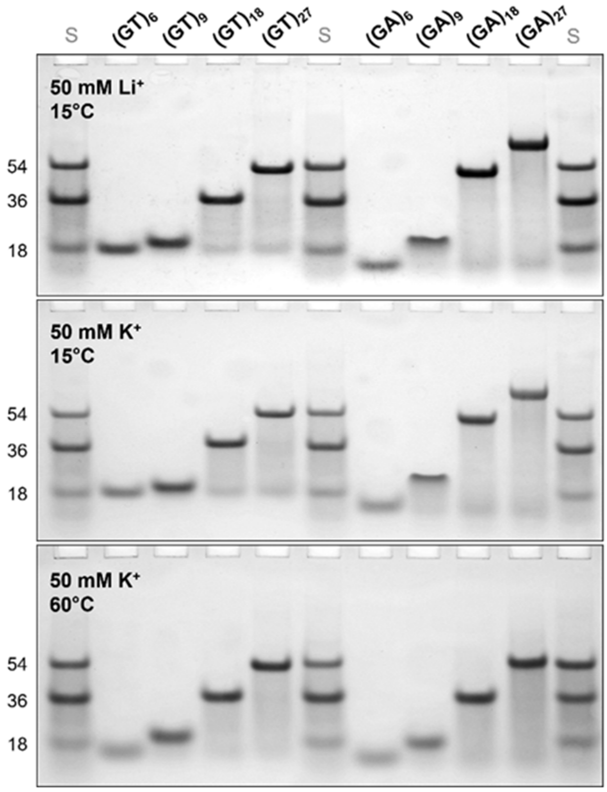

Figure S4. PAGE analysis of $(GT)_n$ and $(GA)_n$ in mBR in the presence of 50 mM LiCl and KCl. Electrophoretic separation was performed in a 12% gel at two different temperatures: 15 and 60 °C, at which DNA occurs in folded and unfolded states.

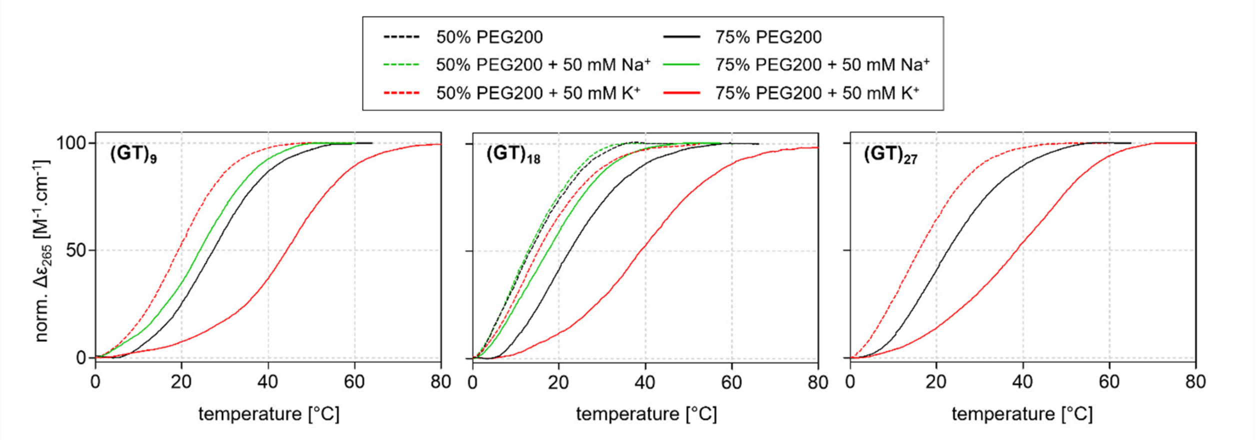

Figure S5. CD melting curves of $(GT)_n$ under different conditions.

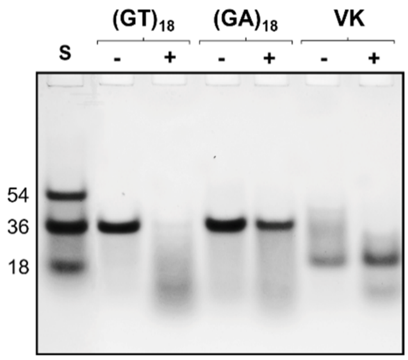

Figure S6. Digestion of $(GT)_{18}$ and $(GA)_{18}$ and VK by Mung Bean nuclease. 5U of enzyme was used for the 45-min reaction at 25 °C. PAGE: 12% in 25 mM mBR, 50 mM KCl pH 7.5, 6 V/cm, 2.5 h at 60 °C.
